# Leading Change and Advancing Health by Enhancing Nurses' and Midwives' Knowledge, Ability and Confidence to Conduct Research through a Clinical Scholar Program in Western Australia

**DOI:** 10.5402/2011/245417

**Published:** 2011-10-25

**Authors:** Rose Chapman, Ravani Duggan, Shane Combs

**Affiliations:** ^1^School of Nursing and Midwifery, Curtin University, GPO Box UWA 1987, Perth 6845, Australia; ^2^Nursing and Midwifery Executive, Joondalup Health Campus, Shenton Avenue, Joondalup, WA 6027, Australia

## Abstract

This paper reports on an evaluation of a Clinical Scholar Program initiated at a hospital in Western Australia. The aim of the program was to build the capacity of nurses and midwives to conduct research and evidence-based practice within the hospital. The program was based on a previous program and consisted of six teaching days and four hours per month release for proposal preparation. At the end of the program participants were asked to complete a short anonymous questionnaire. The answers were analysed using standard processes of qualitative analysis. Themes emerging from the data included program strengths, individual gains, ability to conduct research, and areas for improvement. The findings highlighted that, while the participants considered that they were more knowledgeable and confident to conduct research, they still required support. The Clinical Scholar Program has provided a way to increase the capacity of clinicians to participate in research activities.

## 1. Introduction and Background

Barriers that prevent nurses and midwives from effectively responding to rapidly changing and evolving healthcare systems need to be overcome [1]. To this end educators and administrators are required to provide clinicians with the opportunity and support to lead and diffuse collaborative improvement efforts, lead change to advance health, and engage in lifelong learning [1]. One way to accomplish these outcomes is through providing those nurses and midwives working on the front line with the knowledge and skills to identify practice issues, develop research or quality improvement proposals, implement change, and evaluate the effectiveness of those changes. Therefore, this paper reports on an educational initiative, namely, the Clinical Scholar Program (CSP), undertaken at a nonteaching metropolitan hospital in Western Australia. The aim of the CSP was to further improve the research culture and to build capacity within the hospital of nurses and midwives who have the skills and confidence to engage in research and quality improvement activities. 

The CSP was one outcome of the original collaboration between Joondalup Health Campus (JHC) and Curtin University in 2004 [2]. This collaboration involved the introduction of a Nurse Research Consultancy for one day a week with the aim of implementing and facilitating evidence-based practice within the hospital's Emergency Department (ED). Following the initial success of this initiative, the role was expanded to three days per week servicing the whole hospital and since 2007 until now includes a Nurse/Midwife Research Consultant (N/MRC). The focus of these positions continues to be the facilitation of evidence-based nursing and midwifery practice throughout the hospital. The outcomes from the 2004–2010 collaboration (in terms of publications and presentations) are listed in Table 1. 

Evidence-based practice is a competency standard requirement for both nurses [3] and midwives practicing in Australia [4]. This requirement is supported internationally by both the International Council for Nurses [5] and the International Confederation of Midwives [6]. Undertaking research is part of the scope of nursing [5] and midwifery practice and basic knowledge should include “principles of research, evidence-based practice, clinical interpretation of professional literature, and the interpretation of vital statistics and research findings” ([6], page 3). Given what is already reported in the literature about barriers and facilitators within the clinical setting, getting nurses and midwives engaged in research activities is a challenging process. The literature has highlighted that lack of resources, knowledge, support from the organization, and clinicians own personal characteristics can be barriers to nurses and midwives participating in the research process [2, 7, 8].

The most significant barrier to research utilization within the clinical setting has been shown to be the lack of available necessary resources [7]. A key resource identified by nurses was having a nurse research consultant (NRC) on site that they can approach for help and direction [2]. However, in some instances nurses and midwives are unaware of the available resources to assist them to engage in research [9]. A further barrier preventing nurses and midwives from engaging in research activities has been shown to be their lack of research knowledge, skills, and experience [2, 10]. Often this lack of knowledge is as a result of the type of preservice education received. Older, hospital-based programs usually lack a research component in contrast to education received at undergraduate level [8]. Specifically, these nurses lack the ability to critique research reports and make a determination about the quality of the evidence provided. NRCs were seen as having a role in providing the necessary education that would enable nurses to engage in research activities and be critical consumers of research articles [2]. Provision of appropriate education to help nurses build a foundation in research knowledge and skills within the clinical setting has been perceived as being a facilitator of research [7]. Furthermore, Irish midwives have identified research education as a priority and that further research is necessary to identify how evidence-based practice can be promoted amongst midwives, to increase the number of midwives conducting research and disseminating their findings [11]. 

Chapman and Combs [2] found that while nurses demonstrated a willingness to participate in research activities, they shied away from doing so on their own, preferring a team approach with someone experienced at the helm. In addition, nurses and midwives consider that research should be conducted by specialist researchers and that they should not be expected to lead research projects [9]. A lack of personal motivation was also identified as a reason for nurses [2] and midwives [10] not engaging in research activities. This was compounded by them not seeing any personal benefit in undertaking such activities. In addition, perceptions of not having the authority to bring about changes within the clinical setting, the organisational culture, and lack of support from hospital executive and medical colleagues seemed to add to the barriers [2, 7, 12]. This is supported by Parson and Griffiths [13] who asserted that professional socialisation inherent in the professions of nursing and midwifery is to blame for nurses and midwives obeying authority even in the absence of evidence to support clinical decisions and failing to question traditional practice. A further organizational barrier is the lack of effective systems in place to identify clinicians within the clinical setting who have the potential and enthusiasm to undertake research. As a result of this oversight, those nurses and midwives with good ideas are often overlooked, and as a result their interest in research diminishes [9]. 

Organisational culture is instrumental to nurses and midwives engaging in research activities [2]. To encourage and support clinicians to participate in research activities, leaders within the organisation need to espouse the importance of evidence-based practice and the development of knowledge to support such practice [7, 12]. In addition, clear research-related aims and feedback on research activities from senior staff help to decrease perceived barriers to research [8]. Support structures need to be put in place such as time allotted during work hours for research activities [2]. This strategy is supported by Kajermo et al. [8] who found that, as work schedules became busier, registered nurses did not have the time to read published research and to implement the evidence into practice [7, 12]. Lack of time to participate in research activities has also been shown to be problematic for midwives [9, 10]. Inadequate support through lack of funds to conduct research has been identified as another reason that clinicians do not engage in research within the clinical setting [10].

Opportunities need to be created that enable nurses and midwives to overcome the barriers to research utilisation and production so that they may become agents for change within the health care setting [1]. The hospital where the CSP was initiated has a high level of nondegree prepared (preservice) registered nurses and midwives and very few staff holding formal postgraduate qualifications at diploma level, with even fewer holding High Degree by Research (HDR) qualifications. Therefore, although some nurses and midwives are participating in the research process, the numbers remain very low even after seven years of the NRC and N/MRC being available. Bearing these barriers in mind and to increase the capacity of clinicians working on the floor to conduct research, it was deemed necessary to develop the CSP. This program would also assist in bridging the research gap between university-prepared and hospital-prepared nurses and midwives [1]. The program aimed to help nurses and midwives develop research knowledge and skills so that they would be able to critically appraise research reports, implement findings into clinical practice, develop research ideas from clinical practice, and conduct research studies related to either nursing or midwifery.

## 2. Materials and Methods

### 2.1. The Clinical Scholar Program

The CSP at JHC was based on the one described by Schultz [14]. The CSP was designed to provide scholars with the skills and confidence to identify a clinical problem or need to change practice, review the literature, write a research/QI proposal, conduct the research/QI project, implement change, evaluate the effectiveness of the change, make modifications to the practice as necessary, and disseminate the outcomes within the hospital and to the broader nursing and midwifery community. The program was supported by the Director of Nursing and Midwifery (DONM) who agreed to release staff for the six full days of teaching and provided them with an additional four hours per month project preparation time. 

Once the program had gained support from the hospital executive, an email was sent to all nurses and midwives working within JHC and flyers placed in all wards and departments inviting clinicians to be part of the program. Clinicians were asked to provide evidence as to how they met the selection criteria which, similar to Schultz's [14] program, was those nurses and midwives who had little research experience but possessed a high level of curiosity, questioned current practice, thought critically, and were reflective practitioners. We had eleven applications for the eight places on the program. A review panel was formed to identify and select the successful applicants. Those applicants that were not successful were provided with feedback from the Director of Research-Nursing (DoR-N) that would enable them to work toward selection in the next round.

Participants of the program included seven clinical scholars (CSs) (one of the participants withdrew due to personal issues) and the involvement of four nurse/midwife research mentors. Tables 2 and 3 provide the CSs' demographic characteristics and level and areas of employment. The nurse/midwife research mentors were those nurses/midwives who were currently or who had successfully completed research programs. In the main the mentors had not gained higher degrees by research and, as such, had little formal education regarding the research process. Therefore, a decision was made to include the mentors in the education program. 

The CSP consisted of six full teaching day workshops over nine months in 2010. The teaching days involved two four-hour sessions; the morning session focused on didactic teaching and individual and small group work, and the afternoon session enabled the CSs to work toward developing a research proposal. All of the morning sessions were pedagogical and andragogical in nature, the first four mornings being focused on research methodology and the final two morning sessions provided participants with the theory of project management (see Table 4). 

The objectives of the course were to enable clinicians to:

- demonstrate knowledge of nursing research, evidence-based practice (EPB), and quality improvement;
- demonstrate knowledge of project management;
- apply the principles of mentor-mentee relationships,
- Demonstrate an understanding of new methods for knowledge sharing.
- develop and present a nursing research, EBP, or quality improvement proposal.

In addition to the formal teaching program, participants were expected to complete homework assignments in between workshops in order to reinforce and clarify new skills. On completion of the program, the CSs presented their research proposals to hospital staff, allowing for the opportunity to demonstrate the value of nursing and midwifery research and accomplishment. 

Only four of the seven CSs completed the program. One left after the second workshop because she identified that she was unable to commit enough of her own time to meet the requirement of developing a research proposal. The other two withdrew towards the end of the program, one because of ill health and the other because she did not feel confident in her ability to progress with the research. In addition, two other CSs, who completed the program and developed research proposals and presented to the hospital, left the workplace to pursue other career options in nursing. Therefore, four projects were presented to hospital staff, and of these two will be run in 2012. The CSs will be supported by the DoR-N, N/MRC, and research mentors to complete their projects, aimed at achieving publications, conference presentations, and change in practice. 

On completion of the program and following the presentations to the hospital staff, the participants of the CSP were asked to provide anonymous qualitative feedback on the program. A short questionnaire was administered to the CSs which was comprised of six open-ended questions regarding specific aspects of the program, with a seventh question asking for any additional comments that the participants would like to make. Answers to the questions were analysed using standard processes of qualitative analysis as identified by Speziale-Streubert and Carpenter [15] and included coding, finding categories, clustering, and identifying patterns and meanings. Data were reflected on line by line to identify significant meanings, that is, words or phrases that the participants used which we identified as being of interest or importance [16]. The coded meanings were then categorised and clustered and the relationship between them identified, thus confirming that the themes which emerged were adequate and informative. The following section provides the results of this evaluation.

## 3. Results

The feedback from participants fell into four broad categories, namely, program strengths, individual gains, ability to conduct research, and areas for improvement (Table 5). These categories will be presented together with participants' quotes attesting to their confirmability. 

### 3.1. Program Strengths

Under program strengths the participants focused on the support and commitment that they received from the hospital executive to enable them to undertake the program and the relevance and quality of the teaching materials.

**Support**

When participants were asked about program strengths, they credited the CSP and the hospital for the provision of necessary resources and support to undertake research. For example, one participant stated that *“… access to resources and supportive structure, the notion that clinical research is supported, encouraged and valued here. That research is formalised here” (participant 4). *

**Hospital's Commitment**

Of note they mentioned that this initiative demonstrated the hospital's commitment to encouraging and supporting research within the clinical setting. They also felt that this program helped them to overcome negative preconceptions that they had to engaging in research activities. For this participant the CSP *“made Nursing Research less daunting” (participant 3). *

**Program Materials and Structure**

Participants considered that program teaching and learning materials were relevant to their needs and content was delivered appropriately. *“Presentations and handouts were relevant and of a high quality” (participant 2)*. The small group learning style used made the classroom setting comfortable and allowed participants to get to know one another and learn from and support one another. One participant enjoyed the opportunity of *“… learning from each other …” (participant 5). *

### 3.2. Individual Gains

Participants' feedback revealed that the CSP helped to increase their knowledge and awareness of research. In addition, the program provided leadership opportunities related to research and allowed for a feeling of achievement.

**Knowledge**

Overall the feedback from the participants indicated that they had gained foundational knowledge related to nursing and midwifery research. As one participant wrote, *“it helped me to gain a great base of knowledge about nursing research” (participant 2). *They saw this knowledge as instrumental in assisting them to be able to look more critically at research reports. *“Gave me enough knowledge to begin to question when reading research” (participant 3). *

**Awareness**

The program also helped to make them aware of the value of research and of the research activities that are ongoing at the hospital. For example, *“… awareness of the importance of nursing research, awareness of research happening at JHC, ability to read research articles critically, desire to remain involved with nursing research …” (participant 2). *

**Opportunity**

The CSP enabled participants to have leadership opportunities specific to research and feel more confident with the research process and provided them with *“a thirst to want to do more” (participant 3)*. One participant thought the program had given her *“The opportunity to lead and to try to develop research group. A bit more confidence in the process” (participant 4)*.

**Sense of Achievement and Gratitude**

Many participants were grateful to the hospital for this program and expressed this in their feedback under the question relating to “additional comments.” For example, one participant stated that she *“ thoroughly enjoyed the experience. At times have found it hard to fit in with work/life but feel it has been well worth it. Can't wait to commence the project” (participant 3)*. And another wrote, * “I think it is an innovative model which could be adapted by other organisations in the promotion of nursing research. I feel very pleased to have played a small role in it” (participant 6). *

These comments demonstrate a feeling of achievement and the desire to conduct research. They also show that participants valued the experience and saw the value of the CSP beyond their own hospital.

### 3.3. Ability to Conduct Research

Participants noted feeling more confident to conduct research but had reservations about attempting a research project on their own.

**Research Process**

Participants considered that they had gained a better understanding of the research process as a consequence of the CSP, which resulted in them feeling more confident about conducting research. *“I am much more aware of the process involved in nursing research …” (participant 2)*.

They expressed more confidence and that they would be able to undertake a research project within the clinical setting. For example, this CS wrote* “enough to commence research project with guidance and further study. I now feel it is something I am capable of” (participant 3).*

**Support and Guidance**

They did however feel that they were still at a beginning level and would need further support and guidance when undertaking a research project from someone more experienced than them. Participant five expressed this as *“I believe I have a good grounding to conduct research at a novice level. However I feel I would need further guidance and mentoring through the process to feel confident.” (participant 5)*. Participant 4 concurred with *“still feel will require guidance from mentor but am much clearer on what is required” (participant 4). *

### 3.4. Areas for Improvement

Feedback from participants proved to be very helpful with practical suggestions for how to improve the program. The suggestions focussed on changes to the program structure and to support from the hospital.

**Time**

For some participants the reality of the program demands was in conflict with their expectations. The time commitment required came as a surprise. For example, *“Time frame required was far in excess of what I was prepared for working full time” (participant 1)*.

Participants also found that they had an unrealistic perception of the amount of additional study that was needed in order for them to grasp the material that was covered during the study days. *“It did require a lot of individual study time …” (participant 2).*

This requirement added to the demands on their time at home. It was felt that this expectation needed to be clarified with future scholars prior to the onset of the program.

**Program Delivery**

Some participants felt that the program should be delivered in shorter, more frequent sessions instead of one-day sessions, monthly. It was felt that more frequent sessions would aid learning and that more exercises to encourage and test learning be incorporated. *“Better with more frequent shorter sessions and more opportunity to apply what has been learned. For example, exercises to complete in aspects of the course” (participant 4)*. However, this strategy had the potential to increase demands on their time, which was an issue that was also raised.

**NRC, N/MRC and Mentor Contact**

It was also suggested that individual meetings be arranged so that scholars had the opportunity to meet with NRC, N/MRC, and mentors outside of the classroom setting for guidance on their project ideas. *“Meetings with lecturers and preceptors bi-monthly at personal level” (participant 1). *

It would seem from the feedback that the learning contract approach to the scholar-mentor relationship, with both parties determining the parameters of the interaction, did not work effectively. This resulted in participants feeling that a more prescriptive approach was necessary. *“More rigour to meetings between scholars and mentors; a framework” (participant 6).*

**Development of Research Ideas**

Participants expressed difficulty with identifying research problems that they wanted to investigate as part of the CSP. They suggested that some direction be given to participants prior to the start of the program so that they could begin to review the literature and articulate their research problem at the outset of the program. *“Have participants think about topics before the program begins” (participant 2). *

**Managerial Support**

It appeared from the feedback that departmental managers did not understand the work-release arrangements related to the CSP. This posed problems for the scholars in that they were unable to obtain support at ward level, resulting in them not being freed up as previously agreed to work on their research projects. * “Raise awareness with department managers regarding release from work hours/support” (participant 5). *

Hence, the participants suggested that more be done prior to the onset of the program to make managers aware of the program so that they could provide the necessary ward level support.

## 4. Discussion

As a result of the CSP, we have increased the number of nurses who are research competent and able to mentor new staff. The more clinicians who are able to conduct research and mentor novice researchers, the more research projects will be undertaken to improve practice and patient outcomes [17]. The CSP is a new approach and educational model that could be replicated for adoption nationally and internationally to ensure that clinicians are prepared to respond to escalating information in all practice settings [1]. The program provided participants with new competencies in decision making, quality improvement, and leadership and was one way that the clinicians were able to continue their education in a nonthreatening environment and engage in lifelong learning [1]. Through the CSP we were able to implement three of the eight recommendations from the IOM [1]. The CSP provided clinicians with the skills to lead and diffuse collaborative improvement efforts and conduct research to redesign and improve practice environments (Recommendation 2); ensure that nurses engage in lifelong learning (Recommendation 6); prepare and enable nurses to lead change to advance health and prepare clinicians to assume leadership positions (Recommendation 7) [1]. 

Although the program proved to be successful, we learned several lessons. Only one of our CSs had completed a research unit in their preservice education program. However, on completion of the CSP, our participants considered that they were more knowledgeable about the research process and felt better able to conduct research. This finding is consistent with the literature that has shown lack of knowledge to be a major barrier to nurses and midwives conducting research or applying evidence-based practice in the clinical setting [2, 18, 19]. To enhance this outcome we recommended that the selection criteria for future CSPs take into account previous research education. Direct entry into the CSP will be afforded to those nurses and midwives who have completed a research unit within their preservice education program. This will allow the CSP to build on this basic knowledge and understanding of research. Those candidates who went through a hospital-based training program with no inclusion of research in the curriculum will need to first do a short self-directed course on research before entry into the CSP [8]. This will also serve to ensure that all program participants are at a similar place in their learning and experience at the start of the program, making teaching and learning smoother. 

Support to undertake research was identified as being important to the CSs in this program. Support comprised a few dimensions. Firstly, the program itself signalled the hospital's formal recognition of the importance of nursing and midwifery research. This demonstrated to the CSs that the hospital was committed to the process and to improving nursing and midwifery research output. This in turn had the effect of encouraging the CSs to want to undertake research and assisted them in overcoming any preexisting negative perceptions that they may have had related to research. This finding is supported by Yava et al. [12] who found lack of support from the hospital management to be a barrier to nurses participating in research activities and advocated for increasing support by the organisation and its managers. Hence, commitment and support from the hospital is vital to nurses and midwives having the opportunity to develop research skills and knowledge and feeling motivated to undertake research activities. The CSP demonstrated the hospital's support for nursing and midwifery research in a tangible way, thus helping to positively influence the research culture. 

CSs found the structured approach of the program to be supportive. Having clear program goals and timeframes together with regular feedback is consistent with the recommendations of Kajermo et al. [8] who found that when research goals and feedback on research activities are unclear, they act as a barrier to research. The program also allowed the CSs to have access to resources in the form of the mentors, the DoR-N, and N/MRC and research teaching and learning materials which they found to be of help. This is supported by Chan et al. [19] who found that the introduction of nurse researchers into the clinical setting resulted in clinical nurses engaging in research. Participants in the CSP also developed knowledge and skills to support other colleagues and future CSs to utilise research findings and undertake research in the clinical setting. Thus the program is “self-sustaining” [17, page 99] allowing for the ongoing development of a positive research culture within the hospital. A long-term recommendation for future research would be to study the effect of the CSP on the nursing and midwifery research culture of the hospital. This could be conducted as a longitudinal study investigating nurses and midwives knowledge, attitudes, and use of research and evidence-based practice within the hospital and the wider profession. 

The small group format used for the teaching sessions allowed for the CSs to learn from one another and to feel supported by their peers. This positive experience is likely to be due to the fact that while CSs learnt research and project management theory and skills together for the purpose of developing a research project, they individually worked on their research proposals. Brewer et al. [20] found that group dynamics proved to be difficult to manage and a hindrance to the research process because clinical scholars in their program worked as part of a team. It would be interesting to see if this positive outcome related to small group learning, found in this current program, would continue if CSs were asked to work together on a project as a research team. 

All CSs reported an increase in the knowledge around research theory, research process, and project management. This increased understanding of research also assisted them in critical appraisal of published research and determining quality evidence. With greater understanding of how research is undertaken and evidence is produced, the CSs showed an increased awareness of the value of research within the clinical setting. The CSP also resulted in increased motivation amongst the CSs to conduct research. This motivation was the result of them feeling more confident about undertaking research which was the direct consequence of them being given the opportunity to design and lead a study within their own clinical area. This finding is similar to that of Brewer et al. [20], where clinical scholars showed an increase in confidence to question and feelings of empowerment within the clinical setting. In short, the CSP gave them a feeling of having accomplished something significant within the clinical setting, resulting in them feeling positive towards research and the hospital as a whole. Hence, creating such opportunities within the hospital setting can go a long way to ensuring more satisfied staff who remain with the organisation and contribute to the development of the organisation. Such initiatives stand to also strengthen the hospital by encouraging more nursing and midwifery staff to undertake further formal education based [20] on their feeling of achievement from the CSP. 

Although the CSs mentioned feeling more confident and knowledgeable to undertake research activities, they highlighted the need for ongoing and continued support. Clinicians' participation in research does not occur in isolation and requires organizations to provide personnel and financial support and resources such as computers and adequate space [21]. The nursing executive at JHC is committed to providing staff with the required skills, knowledge, and resources to enable them to conduct research and evidence-based practice within the hospital. The successful CSP has built on the initial introduction of the NRC in 2004 [2] and will continue to increase the capacity of clinicians as regards the knowledge and skills that they require to effectively implement evidence-based practice. 

The literature has highlighted that the time pressure on nurses is a hindrance to research utilization [18, 21]. For the participants in the CSP, this is related to the demands and the commitment they need, to keep up with the requirements of the program. Similar to the participants in Shultz's [14] model, the participants in our CSP were given four hours additional educational leave to work on their proposal development. The program coordinators considered that the expectation that the clinicians would commit time outside of the hours provided by the hospital was made clear at the onset of the program. However, the evaluation demonstrated that the participants considered that a greater emphasis on this requirement would have been helpful. Similar to the nurses in Thompson et al.'s [22] study, the CSP participants experienced cumulative demands on their time including work, home, and family. Richards and Potgieter's [23] study demonstrated that some of the barriers to nurses participating in formal continuing education included, but were not limited to, job and family responsibilities. To overcome this limitation, future CSPs may require hospital executive to provide the participants with additional education hours and more flexible and supportive work schedules [23]. In addition we suggest that clearer articulation of the time demands occurs at the recruitment phase and again at the start of the CSP. Hence, scholars will have a better understanding of what is needed and how the workload will impact their work and personal lives. In addition, a formal contract between the hospital and the CS may be needed with a 50/50 time split, where the CS agrees to match the time provided by the hospital with his or her own personal time. However, an explorative study and evaluation of how the CSs used their time and organised their work release are imperative before any further commitment of hours occurs.

Some of the participants in the CSP noted that they would have preferred if the program was delivered in smaller sessions rather than in blocks and that their learning should have been tested. One interpretation of these comments could be that some of the clinicians would have preferred to have been tested along the way as a motivator to ensure that they studied or completed their assignments in-between workshops. The content and delivery of the program were both pedagogical and andragogical in orientation and although was designed to provide the learner with basic research and project management theory, it still required the participants to be self-directed in their learning. However, participants' individual learning styles were not considered, and it may have been useful to identify these in the beginning of the program using instruments that have already been developed for this purpose such as Kolb's Learning Style Inventory [24]. These tools have been used in nurse education with varying degrees of success [25–27]. As a result of this understanding, the educational program could have been developed to be inclusive of all learning styles. More empirical research needs to be conducted to explore the implications of registered nurses and midwives' learning styles when participating in a CSP. 

In the evaluation of the program the participants noted that they wanted a more directive approach to the mentor/mentee relationships and required the DoR-N and the N/MRC to arrange meetings between them bimonthly. The mentees and mentors were asked to develop contracts between them outlining the mentees learning objective, the timeframe, and the resources required to meet these. The teams were asked to provide the DoR-N with these in an electronic format prior to the second session. However, even after frequent reminders, not all of the mentee/mentor teams complied with the request, and, as the program was coming to an end, the teams identified that they did not have the knowledge in the beginning of the CSP to effectively articulate what it was they needed. The participants of the CSP were provided with a two-hour session in the first workshop that outlined the objectives of the mentor/mentee relationship, mentorship, mentor and mentee characteristics, mentor and mentee roles and responsibilities, the reflective cycle, and the mentor/mentee contract. On reflection and based on feedback provided it would appear that more time should have been taken in the beginning of the program to better develop and nurture these relationships. This should be strengthened by the use of a framework for the relationship giving more structure in terms of frequency and focus of mentor-mentee meetings. Given the difficulties voiced around the mentor-mentee relationship, we recommend that mentors and mentees receive research education in separate forums. This will help to establish the mentors' credibility with the mentees and allow for a smoother working relationship. 

The literature has shown that informal mentor/mentee relationships (developed through unstructured social interactions) can be more effective than formal (dictated by an organization) [28]. However, in an American study conducted by Wanberg et al. [29], investigating predictors and outcomes of mentoring in a formal mentoring program, the authors found that mentor proactivity resulted in more frequent and effective mentoring. Higher levels of mentoring were associated with positive program-related outcomes for both the mentor and mentee. Future CSP should focus on the mentor/mentee relationship and provide both partners with the support to develop and continue these important relationships. In addition, it may be beneficial to enable mentees to identify their own mentor rather than the program coordinators arranging these relationships. Further empirical research is needed to evaluate the effectiveness of informal or formal mentor/mentee relationships to the success of nursing and midwifery research. 

Although the hospital executive was extremely supportive of the program, it would appear that ward and department managers did not have a clear understanding of the work-release arrangements provided to the CSP participants. As a result the CSs did not always take their four hours per month proposal preparation time. Future programs will require more proactive dissemination of the requirements and time release of clinicians participating in the program. It is essential that this strategy is undertaken by both the hospital executive and program coordinators and could be conducted face to face during management and ward meetings, by flyers posted in all wards and departments and the hospital newsletter.

## 5. Limitations

This paper has provided the results of an evaluation of a CSP conducted by a small number of clinicians in one hospital in Western Australia. Although the CSs were supportive of the program and could see the benefits of participating, further evaluation of similar programs within this hospital and other sites is essential. The attrition rate of CSs due to reasons mentioned earlier made evaluating the program difficult as it served to reduce the number of participants available to provide feedback. However, given that this was the first such program at JHC, the information that we gleaned from participants helped us in our exploration of the effect of the CSP. Valid and reliable instruments should be developed to measure the effectiveness of implementing these courses including the costs and benefits from the perspective of the agency, participants, and patients.

## 6. Conclusion

The implementation of the CSP was shown to be one way in which the hospital could build research capacity amongst clinicians. It provided nurses and midwives with the opportunity to develop skills that could foster their partnership with other members of the health care team in health care redesign initiatives [1]. Postevaluation of the program demonstrated that the CSs considered that they were more knowledgeable and confident to conduct research in the future. However, the CSs also identified that they only had beginning skills and that they still required support from the DoR-N and N/MRC to complete their research projects. The program participants acknowledged that the hospital executive had created an environment that valued them as clinicians and demonstrated that research was an important aspect of patient/client care. The participants mentioned that they found the time commitment required to complete the research proposal to be problematic and future programs needed to be more upfront about this requirement. The evaluation has provided the hospital and the program coordinators with a clear understanding of what worked in this initial program and how we can make it better into the future.

## Figures and Tables

**Table 1:** Summary of collaboration outcomes 2004–2010.

Year	Publications—peer reviewed	International and national conference presentations
2004	0	2
2005	1	12
2006	2	10
2007	3	4
2008	1	3
2009	2	2
2010	2	3

Total	11	36

**Table 2:** Demographic characteristics of participants of the Clinical Scholar Program.

Nurse demographics	Range	Mean
Age	29–59	46.36
Years after registration	8–38	23.33
Years in current position	2–9	4.6
Years in current setting	2–13	8.22

**Table 3:** Level and areas of employment of clinical scholar participants.

Level	Number
Registered nurse	1
Clinical nurse	2
Clinical nurse specialist	2
Nurse unit manager	1
Staff development nurse	4
Other	1

Work areas	

Aged care	1
Birth suite	1
Education unit	2
Emergency department	2
Maternity	1
Intensive care unit	1
Operating theatres	2
Orthopaedics	1

**Table 4:** Summary of Clinical Scholar Program.

Workshop	Morning topics	Afternoon topics
1	Getting to know you	Reading journal articles
	Effective mentor/mentee relationships	How to critique
	Introduction to research	Start documenting research ideas and bring to the next session
2	Problem statement	Proposal development
	Hypothesis	
	Literature review/critique	
	Quantitative/qualitative design	
3	Quantitative/qualitative sampling and data collection	Proposal development
	Descriptive and inferential	
	statistics Qualitative data analysis	
4	Reliability/validity/ trustworthiness	Proposal development
	Ethics	
	Budget/time line	
5	Project management introduction	Proposal development
	Project scope management	
6	Project cost management	Proposal development
	Human resource management	
	Project communication management	

**Table 5:** Summary of evaluation of CSP.

Categories	Sub categories
	Support
Program strength	Hospital's commitment
	Program materials and structure
	Knowledge
Individual gains	Awareness
	Opportunity
	Sense of achievement and gratitude
Ability to conduct research	Research process
	Support and guidance
	Time
	Program delivery
Areas for improvement	NRC and mentor's contact
	Development of research ideas
	Managerial support
